# Electronic media use and academic performance in late childhood: A longitudinal study

**DOI:** 10.1371/journal.pone.0237908

**Published:** 2020-09-02

**Authors:** Lisa K. Mundy, Louise Canterford, Monsurul Hoq, Timothy Olds, Margarita Moreno-Betancur, Susan Sawyer, Silja Kosola, George C. Patton

**Affiliations:** 1 Murdoch Children’s Research Institute, Melbourne, Victoria, Australia; 2 Department of Paediatrics, The University of Melbourne, Melbourne, Victoria, Australia; 3 Alliance for Research in Exercise, Nutrition and Activity (ARENA), University of South Australia, Adelaide, South Australia, Australia; 4 Integrated Health and Social Welfare Unit, New Children’s Hospital, Pediatric Research Center and University of Helsinki, Helsinki, Finland; Weill Cornell Medical College in Qatar, QATAR

## Abstract

**Introduction:**

The effects of electronic media use on health has received much attention but less is known about links with academic performance. This study prospectively examines the effect of media use on academic performance in late childhood.

**Materials and methods:**

1239 8- to 9-year-olds and their parents were recruited to take part in a prospective, longitudinal study. Academic performance was measured on a national achievement test at baseline and 10–11 years of age. Parents reported on their child’s duration of electronic media use.

**Results:**

After control for baseline reading, watching more than two hours of television per day at 8–9 years of age predicted a 12-point lower performance in reading at 10–11 years, equivalent to the loss of a third of a year in learning. Using a computer for more than one hour a day predicted a similar 12-point lower numeracy performance. Regarding cross-sectional associations (presumed to capture short-term effects) of media use on numeracy, after controlling for prior media exposure, watching more than two hours of television per day at 10–11 years was concurrently associated with a 12-point lower numeracy score and using a computer for more than one hour per day with a 13-point lower numeracy performance. There was little evidence for concurrent effects on reading. There was no evidence of short- or long-term associations between videogame use and academic performance.

**Discussion:**

Cumulative television use is associated with poor reading and cumulative computer use with poorer numeracy. Beyond any links between heavy media use and health risks such as obesity, physical activity and mental health, these findings raise a possibility of additional risks of both television and computer use for learning in mid-childhood. These findings carry implications for parents, teachers and clinicians to consider the type and timing of media exposure in developing media plans for children.

## Introduction

Electronic media use has become the most popular leisure-time activity for children and adolescents (7–18 year olds) [1]. Electronic media use includes screen-based activities such as computer and smart phone use, electronic video games and television viewing. The extent to which electronic media may affect the lives of children is hotly debated [2]. In systematic reviews, electronic media use during childhood and adolescence is linked with physical health risks, such as obesity and poor sleep [3–5] while benefits include access to information, improved technological skills and greater social connection [6]. Although there have been some suggestions that media use may affect learning, the relationship with academic performance is not clear [1, 2].

Given education’s role in later life health and wellbeing [7], understanding the effects of media use on academic performance is important [8]. The mid-primary school years are a time when academic difficulties are often first evident [9, 10] and predictive of later academic failure and school dropout [11]. These middle years are also a time when children’s media use dramatically rises and children have growing autonomy over the media they consume [12].

Electronic media use might affect academic performance in primary school in several ways. It may displace other activities, such as physical activity, sleep or homework, [13, 14] all of which have been positively associated with academic performance [15–17]. Electronic media use has the potential to diminish concentration [18] and children and adolescents may be particularly susceptible to what they see on screen, which in turn may influence their beliefs and behaviours [19]. Excessive media use may even affect brain structure and function including reward processing, as shown in imaging studies with children and adolescents from 5 to 18 years of age [20–23]. However, electronic media use may also enhance school performance through increased access to information and resources, and improved skills in technology.

Existing research suggests some types of media may have a positive effect on academic performance. For example, the benefits of television programs such as Sesame Street have been consistently demonstrated [24]. Computer use has also been linked with better learning outcomes [25, 26] but the evidence is mixed [27]. Some types of video gaming may improve attention skills [28], but other studies suggest video games negatively affect academic performance [29], whilst some large studies in adolescents have found no association [30]. Thus, there is a need to consider the type of media being consumed, not just overall screen time.

Previous research has been limited by a focus on only one or two types of media use, which may explain the inconsistency in patterns previously observed. Television viewing has been frequently studied but much less is known about the effect of newer media, such as video games and the internet. It is possible that the associations observed in the past may be less relevant in today’s media environment where technology is more ubiquitous, varied and there is a convergence of different media types in different devices [31, 32]. Many earlier studies have examined cohorts born several decades ago [3], but patterns of media use are changing rapidly [33]. Importantly, there has been little focus on the timing of exposure, despite evidence that younger children may be more susceptible to the effects of media [34] and that cognitive capabilities at different stages may determine how children use and interact with media [19]. Also, in older adolescents, past use is likely to be an important confounder, yet most studies have focused on older adolescents [35, 36].

Most existing studies have used global measures of academic performance [3, 37], but patterns of association may differ according to the domain of academic performance measured. For example, television viewing has been associated with poorer performance in mathematics but not reading achievement [38]. Therefore, when examining links between media use and learning, the domain of learning may be an important consideration. Brief teacher or even student self-report global measures of academic performance have been frequently examined in previous research [31, 39], but no studies have examined academic performance across multiple domains using national tests of achievement. Finally, very few longitudinal studies have examined the relationship between electronic media use and academic performance and furthermore, most studies have failed to control for prior academic performance [1]. This is important because children with poorer academic performance may use electronic media more than their academically successful peers.

This paper aimed to examine the long-term, cumulative effect of media use on academic performance, as well as looking at the effect of current (cross-sectional associations of) media use, using a standardised national test of achievement in a large population sample during late primary school. Electronic media use was measured across television, computer use and video games. Analyses were adjusted for age, sex and socioeconomic status (SES). Our analyses also included control for baseline academic performance. When examining associations between concurrent media use and academic performance, we control for both baseline media use and academic performance, which allows an estimate of the effect of concurrent media regardless of prior media use. In secondary analyses we controlled for variables known to be associated with both screen time and academic performance, namely emotional and behavioural problems [40, 41] and body mass index [42, 43].

## Material and methods

### Study population and design

Data for this study were drawn from waves one (conducted in 2012), two (2013) and three (2014) of the Childhood to Adolescence Transition Study (CATS). This is a longitudinal cohort study with a broad focus on health, education and social adjustment as children transition through puberty. The full study design is reported elsewhere [44]. Briefly, all grade three children (8–9 years; fourth year of formal schooling) were invited to take part from a stratified random sample of 43 primary schools (Government, Catholic, Independent strata) in metropolitan Melbourne, Australia. A total of 101 schools were approached to take part and the main reason for non-recruitment was schools were too busy. Of the 2289 invited children, 1239 (54%) were recruited through the provision of active, informed parent consent. The main reason for non-recruitment was parents failing to return the consent form.

### Procedure

Research assistants visited each school annually and completed the child assessment for those children with parental consent. Parents completed two questionnaires at wave one; part one was a short questionnaire administered as a paper version at the same time as the consent process, and part two was sent to parents after the child’s data collection session, either as a paper version or online. Parents also completed a questionnaire at each subsequent wave. Teachers completed a short questionnaire for each child at each wave. Ethics approval was granted by the Royal Children’s Hospital Human Research Ethics Committee (#31089). Permission was granted from the Victorian Department of Education and Training and Catholic Education Melbourne to recruit through their schools.

### Measures

#### Academic performance

Academic performance was assessed at waves one and three (Grades 3 and 5) using the National Assessment Programme–Literacy and Numeracy (NAPLAN). NAPLAN measures academic performance across four domains—reading, writing, numeracy, and language conventions (spelling, grammar and punctuation). It is administered to all students in Australian schools in grades three, five, seven and nine. This paper focuses on the reading and numeracy domains, as these are the most reliable domains. A score ranging from 0 to 1000 is provided for each domain completed by each child, which is scaled across all grades [45]. Grade three (8–9 years) students with a score lower than 270 are considered to perform below the national minimum standard and at grade five (10–11 years) the national minimum standard is 374. As a guide, one year of learning between grades three and four (9–10 years) equates to approximately 40 NAPLAN points [45]. NAPLAN data were provided by the Victorian Curriculum and Assessment Authority for children whose parents had provided additional optional consent at recruitment for data linkage (1,153 (93%) parents provided consent).

#### Electronic media use

Electronic media use was measured at grades three and five through parent report on items adapted from the Longitudinal Study of Australian Children. Parents were asked to report how many hours their child spends: a) watching TV or DVDs (on TV or computer), b) playing videogames (on computer or console (e.g. Xbox)), and c) using the computer (email/schoolwork/internet access/chat). These questions were repeated for school days and weekend days. To calculate average hours spent per day using each type of media, the total weekday number of hours was multiplied by five and added to the total weekend hours multiplied by two, with the sum then divided by seven. Categorical variables were derived for each electronic media type based on the distribution of data and in line with existing studies: TV (‘ ≥ 0 and ≤ 1 hour’ versus ‘> 1 and ≤ 2 hours’ versus ‘> 2 hours’); video games and computer use (‘none’ versus ‘> 0 and ≤ 1 hour’ versus ‘> 1 hour’).

#### Covariates

To control for confounding the following variables, chosen *a priori* on theoretical grounds, were included in analyses: sex, child’s age (at grade 3) and family SES (at grade 3) [46]. In a second set of models, a measure of behavioural and emotional symptoms (Strengths and Difficulties Questionnaire (SDQ) total score) was included, and in a third set of models body mass index (BMI) z-score was included.

Family SES was calculated for home postcode (parent report) using the Index of Relative Socio-economic Advantage and Disadvantage (IRSAD; population mean (M) = 1000, standard deviation (SD) = 100) from the Australian Bureau of Statistics Socio-Economic Index for Areas (SEIFA). Behavioural and emotional symptoms were measured using parent report on the total difficulties scale of the SDQ [47]. The SDQ consists of 25 items covering five scales: emotional symptoms, conduct problems, hyperactivity/inattention, peer relationship problems and prosocial behaviour. A total difficulties score is derived from the first four subscales and is a marker of overall mental health problems. Parents rate each of the items as “Not True”, “Somewhat True” or “Certainly True”. “Somewhat True” is always scored as one but the scoring of “Not True” and “Certainly True” varies by item as zero or two. The total difficulties score can range from 0 to 40 with higher scores indicating higher levels of problems. Height and weight were measured by trained staff. BMI (kg/m^2^) was calculated and transformed to z-scores (according to the WHO growth standards [48]).

#### Auxiliary variables

Further variables were included in the multiple imputation model to handle missing data (see data analysis section). These auxiliary variables were selected as they had moderate/high correlation with those to be imputed [49]. The following variables (all measured at grade 4) were included: average hours spent per day watching TV (three level categorical variable), average hours spend per day playing videogames (three level categorical variable), average hours per day spent using a computer (three level categorical variable), SDQ total difficulties score (parent report), and child BMI z-score. School sector (Government, Catholic, Independent) at grade 3 was also included, along with overall ratings of children’s abilities in English and mathematics provided by teachers at grade 3.

### Data analysis

Child characteristics, academic performance and media use were summarised; means and standard deviations were calculated for all continuous measures, and percentages were calculated for categorical variables. Linear generalised estimating equations (exchangeable correlation structure within school at grade 5 and robust standard errors) were used to investigate the effect of electronic media use (watching TV, or playing videogames, or using a computer) on NAPLAN scores (numeracy, reading) at grade 5 with the effects of each electronic media type examined in separate models.

Specifically, we performed both cross-sectional (short-term effects) and longitudinal (long-term effects) analyses for each media type and NAPLAN domain. To examine the long-term effect of media use on academic performance, three models were fitted in which the categorical exposure was grade 3 media use. Firstly, models were adjusted for child sex, child age (in years) (grade 3), SES (grade 3), and corresponding NAPLAN score (reading or numeracy) at grade 3. A second set of models was fitted, adjusting for all previously mentioned confounders, with the addition of SDQ total score at grade 3. Finally, a third set of models was fitted, adjusting for all previously mentioned confounders, with the addition of BMI z-score at grade 3. Similarly, we built models for cross-sectional analyses, but this time the exposure was grade 5 media use. We then built additional models for cross-sectional analyses, with the exposure being grade 5 media use, and with adjustment for grade 3 media use.

There were missing data in all variables except child sex, child age, and SEIFA IRSAD. We used multiple imputation to deal with potential missing data biases, imputing 20 complete data sets using chained equations [50]. Linear regression was used to impute the continuous variables and ordinal logistic regression to impute ordinal variables, in each case including all other analysis (see below) and auxiliary (see above) variables as predictors (see S1 Table).

## Results

Of the 1239 children recruited into the study at grade 3, slightly less than half were males (n = 572 (46.2%)), and the majority were born in Australia (n = 1055 (87.8%), missing n = 37). The average age of the cohort was 9.0 years (SD = 0.4) at grade 3 and 10.9 years (SD = 0.4) at grade 5. At baseline, just over 60% were in the top two most advantaged SES quintiles (1^st^ quintile (most advantaged): n = 423 (34.1%); 2^nd^ quintile: n = 346 (27.9%); 3^rd^ quintile: n = 194 (15.7%); 4^th^ quintile: n = 109 (8.8%); 5^th^ quintile (most disadvantaged): n = 167 (13.5%)). Nearly three in four children went to a Government school in grade 3 (n = 893 (72.1%), and two in three children went to a Government school in grade 5 (n = 849 (70.3%), missing n = 30).

Table 1 summarises the academic performance, media use and other characteristics of the 1239 children at grades 3 and 5. NAPLAN results in the analysis sample aligned well with results for the Victoria metropolitan area presented in national reports (grade 3 reading 436.2 vs. 440.8 in CATS; grade 3 numeracy 412.8 vs. 418.6 in CATS; grade 5 reading 512.7 vs. 521.5 in CATS; grade 5 numeracy 500.3 vs. 503.5 in CATS) [45, 51]. Rates of media use were the same in grades 3 and 5 for TV (grade 3 1.9 hrs/day; grade 5 1.9 hrs/day) with small increases for video games (grade 3 0.7 hrs/day; grade 5 0.9 hrs/day) and computer use (grade 3 0.7 hrs/day; grade 5 1.0 hrs/day). The proportion of children watching TV in each of the three categories of average daily duration were similar in grades 3 and 5. One in five children did not play videogames in grade 3, whereas in grade 5 this proportion increased to one in four children. Just over one-quarter played more than one hour of videogames per day in grade 3, a proportion that increased to nearly one-third in grade 5. Computer use increased between grades 3 and 5. Nearly one-quarter of students did not spend time using computers in grade 3, whereas in grade 5 just over one in ten children did not spend time using computers. Nearly one in five children used computers for more than one hour per day in grade 3, a proportion that had nearly doubled in grade 5.

**Table 1:** Summary of academic performance, media use, BMI z-score and SDQ total score amongst n = 1239 children, stratified by school year level.

	Grade 3		Grade 5	
	n^a^	Value	n^a^	Value
Academic performance (mean (SD))				
NAPLAN–Reading score	1067	440.8 (86.1)	1035	521.5 (77.5)
NAPLAN–Numeracy score	1053	418.6 (72.4)	1035	503.5 (68.0)
Media use (hours/day) (n (%))				
Watching TV	919		882	
≥ 0 and ≤ 1 hour		180 (19.6)		180 (20.4)
> 1 and ≤ 2 hours		366 (39.8)		370 (42.0)
> 2 hours		373 (40.6)		332 (37.6)
Playing video games	920		849	
0 hours		192 (20.9)		204 (24.0)
> 0 and ≤ 1 hour		498 (54.1)		381 (44.9)
> 1 hour		230 (25.0)		264 (31.1)
Using a computer	920		847	
0 hours		219 (23.8)		98 (11.6)
> 0 and ≤ 1 hour		549 (59.7)		497 (58.7)
> 1 hour		152 (16.5)		252 (29.8)
BMI z–score (mean (SD))	1186	0.6 (1.2)	1094	0.5 (1.2)
SDQ total score (mean (SD))	1204	8.4 (5.5)	902	8.3 (5.9)

BMI, Body Mass Index; NAPLAN, National Assessment Programme–Literacy and Numeracy; SDQ, Strengths and Difficulties Questionnaire.

^a^ Number with observed data (remainder are missing).

Table 2 summarises the association of daily time spent using electronic media in grade 3 with NAPLAN reading and numeracy scores at grade 5 adjusting for various confounders, as well as baseline academic performance. The findings suggest that children who watched more than 2 hours of TV per day in grade 3 on average had a lower grade 5 NAPLAN reading score (when adjusted for child sex, child age, SES, and baseline academic performance) compared with those who watched 1 hour or less (β = -12.5, 95% CI: -24.1 to -0.8). This represents a delay of approximately 4 months in learning. Smaller negative effects were observed for watching TV for > 1 hour to ≤ 2 hours per day at grade 3 on grade 5 NAPLAN reading (β = -7.2, 95% CI = -18.2 to 3.8) and numeracy scores (β = -8.1, 95% CI = -17.2 to 1.1), and also for watching more than 2 hours per day at grade 3 on grade 5 NAPLAN numeracy scores (β = -6.1, 95% CI: -15.9 to 3.7).

**Table 2:** Effect (mean difference) of media use at grade 3 on NAPLAN scores at grade 5 amongst n = 1239 primary school children, controlling for baseline academic performance (multiple imputation analysis).

	Model set 1^a^			Model set 2^b^			Model set 3^c^		
	β	95% CI	p	β	95% CI	p	β	95% CI	p
**Reading**									
TV									
≥ 0 and ≤ 1 hour	ref	ref	ref	ref	ref	ref	ref	ref	ref
> 1 and ≤ 2 hours	-7.2	-18.2 to 3.8	0.20	-7.1	-18.0 to 3.8	0.20	-7.2	-18.2 to 3.9	0.20
> 2 hours	-12.5	-24.1 to -0.8	0.04	-12.1	-23.6 to -0.6	0.04	-12.2	-23.9 to -0.6	0.04
Video Games									
0 hours	ref	ref	ref	ref	ref	ref	ref	ref	ref
> 0 and ≤ 1 hour	-0.3	-8.3 to 7.7	0.95	-0.9	-8.9 to 7.1	0.83	-0.8	-8.9 to 7.2	0.84
> 1 hour	3.4	-7.3 to 14.1	0.54	4.1	-6.6 to 14.7	0.45	4.2	-6.5 to 14.9	0.44
Computer									
0 hours	ref	ref	ref	ref	ref	ref	ref	ref	ref
> 0 and ≤ 1 hour	-1.6	-10.6 to 7.3	0.72	-2.0	-10.9 to 6.8	0.65	-2.1	-11.0 to 6.8	0.64
> 1 hour	-7.9	-19.5 to 3.7	0.18	-8.1	-19.6 to 3.4	0.16	-8.3	-19.8 to 3.2	0.16
**Numeracy**									
TV									
≥ 0 and ≤ 1 hour	ref	ref	ref	ref	ref	ref	ref	ref	ref
> 1 and ≤ 2 hours	-8.1	-17.2 to 1.1	0.08	-7.9	-16.9 to 1.2	0.09	-7.9	-16.9 to 1.2	0.09
> 2 hours	-6.1	-15.9 to 3.7	0.22	-5.7	-15.6 to 4.2	0.26	-5.6	-15.5 to 4.3	0.26
Video Games									
0 hours	ref	ref	ref	ref	ref	ref	ref	ref	ref
> 0 and ≤ 1 hour	2.1	-4.6 to 8.8	0.54	1.6	-4.8 to 8.1	0.62	1.6	-4.8 to 8.1	0.62
> 1 hour	3.8	-6.0 to 13.7	0.45	4.7	-4.9 to 14.4	0.33	4.7	-5.0 to 14.5	0.34
Computer									
0 hours	ref	ref	ref	ref	ref	ref	ref	ref	ref
> 0 and ≤ 1 hour	-4.7	-12.3 to 3.0	0.23	-5.1	-12.5 to 2.4	0.18	-5.1	-12.5 to 2.3	0.18
> 1 hour	-12.6	-23.4 to -1.7	0.02	-12.9	-23.5 to -2.3	0.02	-12.9	-23.5 to -2.3	0.02

BMI, Body Mass Index; CI, confidence interval; NAPLAN, National Assessment Programme–Literacy and Numeracy; SDQ, Strengths and Difficulties.

^a^ Adjusted for child sex, child age (in years) (grade 3), Socio-Economic Indexes for Areas (SEIFA) advantage/disadvantage quintile (grade 3) and corresponding NAPLAN score (reading or numeracy) (grade 3).

^b^ Adjusted for child sex, child age (in years) (grade 3), Socio-Economic Indexes for Areas (SEIFA) advantage/disadvantage quintile (grade 3), SDQ total score (grade 3), and corresponding NAPLAN score t(reading or numeracy) (grade 3)

^c^ Adjusted for child sex, child age (in years) (grade 3), Socio-Economic Indexes for Areas (SEIFA) advantage/disadvantage quintile (at grade 3), SDQ total score (at grade 3) and BMI z-score (at grade 3), and corresponding NAPLAN score (reading or numeracy) (grade 3).

Children who used a computer for more than 1 hour per day compared with those who did not spend time using a computer, on average had a lower NAPLAN numeracy score (β = -12.6, 95% CI: -23.4 to -1.7, an approximate 4 month delay in learning). Smaller negative effects were observed for using a computer for 1 hour or less at grade 3 on grade 5 reading (β = -1.6, 95% CI = -10.6 to 7.3) and numeracy (β = -4.7, 95% CI = -12.3 to 3.0) scores at grade 5, and for using a computer for more than 1 hour at grade 3 on grade 5 reading scores (β = -7.9, 95% CI = -19.5 to 3.7).

Associations between time spent playing video games at grade 3 and NAPLAN reading or numeracy scores at grade 5 were very small.

Effects were similar when further adjusted for SDQ total score (a measure of behavioural and emotional problems; Table 2, model set 2) and BMI z-score (Table 2, model set 3).

Table 3 summarises the association of daily time spent using electronic media in grade 5 on NAPLAN reading and numeracy scores at grade 5. Children who watched between 1–2 hours of TV per day and those who watched more than two hours per day in grade 5 on average had a lower grade 5 NAPLAN reading score compared with those who watched 1 hour or less (β = -9.4, 95% CI: -19.0 to 0.3; β = -10.8, 95% CI = -20.4 to -1.3, respectively (a delay of approximately 3 months)). These effects were consistent when adjusted further for SDQ total score (Table 3, model set 2) and BMI z-score (Table 3, model set 3). There was evidence of an association between watching more than 2 hours of TV per day at grade 5 and lower grade 5 NAPLAN numeracy scores (β = -12.4, 95% CI: -21.6 to -3.1), a delay of approximately 4 months. There was also evidence to suggest an association between using a computer for more than 1 hour per day, compared with no computer use, and lower grade 5 NAPLAN numeracy scores (β = -13.3, 95% CI: -23.8to -2.8), a delay of approximately 4 months. These effects were consistent when further adjusted (Table 3, model sets 2 and 3). There was no evidence to suggest playing video games at grade 5 was associated with either NAPLAN reading or numeracy scores at grade 5.

**Table 3:** Effect (mean difference) of media use at grade 5 on NAPLAN scores at grade 5 amongst n = 1239 primary school children, controlling for baseline academic performance (multiple imputation analysis).

	Model set 1^a^			Model set 2^b^			Model set 3^c^		
	β	95% CI	p	β	95% CI	p	β	95% CI	p
**Reading**									
TV									
≥ 0 and ≤ 1 hour	ref	ref	ref	ref	ref	ref	ref	ref	ref
> 1 and ≤ 2 hours	-9.4	-19.0 to 0.3	0.06	-9.8	-19.5 to -0.2	0.05	-9.9	-19.5 to -0.2	0.05
> 2 hours	-10.8	-20.4 to -1.3	0.03	-11.4	-20.7 to -2.0	0.02	-11.6	-21.1 to -2.1	0.02
Video Games									
0 hours	ref	ref	ref	ref	ref	ref	ref	ref	ref
> 0 and ≤ 1 hour	-0.7	-9.2 to 7.8	0.88	-0.9	-9.2 to 7.4	0.83	-0.9	-9.3 to 7.5	0.83
> 1 hour	-3.3	-13.6 to 7.0	0.52	-3.0	-13.2 to 7.2	0.56	-3.0	-13.2 to 7.2	0.56
Computer									
0 hours	ref	ref	ref	ref	ref	ref	ref	ref	ref
> 0 and ≤ 1 hour	-0.1	-12.1 to 12.0	0.99	-0.7	-12.7 to 11.3	0.91	-0.7	-12.7 to 11.3	0.90
> 1 hour	-6.5	-18.6 to 5.6	0.29	-7.0	-18.9 to 4.9	0.25	-7.0	-18.9 to 4.8	0.24
**Numeracy**									
TV									
≥ 0 and ≤ 1 hour	ref	ref	ref	ref	ref	ref	ref	ref	ref
> 1 and ≤ 2 hours	-5.5	-13.7 to 2.8	0.19	-5.9	-13.9 to 2.1	0.15	-5.9	-13.9 to 2.1	0.15
> 2 hours	-12.4	-21.6 to -3.1	0.009	-12.8	-21.9 to -3.7	0.006	-12.8	-21.9 to -3.8	0.006
Video Games									
0 hours	ref	ref	ref	ref	ref	ref	ref	ref	ref
> 0 and ≤ 1 hour	-1.2	-8.6 to 6.3	0.76	-1.4	-8.7 to 5.9	0.71	-1.4	-8.7 to 5.9	0.71
> 1 hour	1.0	-8.1 to 10.2	0.82	1.5	-7.5 to 10.5	0.74	1.5	-7.6 to 10.6	0.74
Computer									
0 hours	ref	ref	ref	ref	ref	ref	ref	ref	ref
> 0 and ≤ 1 hour	-3.2	-12.5 to 6.1	0.50	-4.0	-13.3 to 5.2	0.39	-4.0	-13.3 to 5.3	0.39
> 1 hour	-13.3	-23.8 to -2.8	0.01	-13.9	-24.3 to -3.4	0.01	-13.9	-24.3 to -3.4	0.01

BMI, Body Mass Index; CI, confidence interval; NAPLAN, National Assessment Programme–Literacy and Numeracy; SDQ, Strengths and Difficulties.

^a^ Adjusted for child sex, child age (in years) (grade 3), Socio-Economic Indexes for Areas (SEIFA) advantage/disadvantage quintile (grade 3) and corresponding NAPLAN score (reading or numeracy) (grade 3).

^b^ Adjusted for child sex, child age (in years) (grade 3), Socio-Economic Indexes for Areas (SEIFA) advantage/disadvantage quintile (grade 3), SDQ total score (grade 3) and corresponding NAPLAN score (reading or numeracy) (grade 3).

^c^ Adjusted for child sex, child age (in years) (grade 3), Socio-Economic Indexes for Areas (SEIFA) advantage/disadvantage quintile (grade 3), SDQ total score (grade 3), BMI z-score (grade 3) and corresponding NAPLAN score (reading or numeracy) (grade 3).

Table 4 summarises the association of electronic media use in grade 5 with NAPLAN numeracy and reading scores at grade 5, adjusting for baseline academic performance and baseline media use. All of the associations observed in Table 3 with regards to NAPLAN reading scores were no longer apparent after adjusting for grade 3 media use. However, the association between grade 5 TV watching and grade 5 NAPLAN numeracy score, and the association between grade 5 computer use and grade 5 NAPLAN numeracy score were still evident, suggesting these are proximal; effects.

**Table 4:** Effect (mean difference) of media use at grade 5 on NAPLAN scores at grade 5 amongst n = 1239 primary school children, controlling for baseline academic performance and baseline media use (multiple imputation analysis).

	Model set 1^a^			Model set 2^b^			Model set 3^c^		
	β	95% CI	p	β	95% CI	p	β	95% CI	p
**Reading**									
TV									
≥ 0 and ≤ 1 hour	ref	ref	ref	ref	ref	ref	ref	ref	ref
> 1 and ≤ 2 hours	-7.1	-17.8 to 3.5	0.19	-7.8	-18.5 to 2.9	0.15	-7.8	-18.5 to 2.9	0.15
> 2 hours	-6.7	-17.6 to 4.3	0.23	-7.5	-18.4 to 3.4	0.18	-7.7	-18.7 to 3.2	0.17
Video Games									
0 hours	ref	ref	ref	ref	ref	ref	ref	ref	ref
> 0 and ≤ 1 hour	-0.9	-9.7 to 7.9	0.84	-1.1	-9.7 to 7.5	0.80	-1.1	-9.7 to 7.5	0.80
> 1 hour	-4.7	-15.5 to 6.1	0.39	-4.6	-15.3 to 6.1	0.40	-4.6	-15.3 to 6.0	0.39
Computer									
0 hours	ref	ref	ref	ref	ref	ref	ref	ref	ref
> 0 and ≤ 1 hour	0.3	-11.6 to 12.3	0.96	-0.3	-12.2 to 11.7	0.97	-0.3	-12.2 to 11.7	0.96
> 1 hour	-5.4	-17.5 to 6.8	0.38	-5.7	-17.7 to 6.3	0.35	-5.8	-17.8 to 6.2	0.34
**Numeracy**									
TV									
≥ 0 and ≤ 1 hour	ref	ref	ref	ref	ref	ref	ref	ref	ref
> 1 and ≤ 2 hours	-4.6	-13.3 to 4.1	0.30	-5.2	-13.7 to 3.3	0.23	-5.2	-13.7 to 3.2	0.22
> 2 hours	-12.3	-22.7 to -1.9	0.02	-13.1	-23.3 to -2.8	0.01	-13.1	-23.3 to -2.9	0.01
Video Games									
0 hours	ref	ref	ref	ref	ref	ref	ref	ref	ref
> 0 and ≤ 1 hour	-1.6	-9.2 to 6.0	0.68	-1.8	-9.4 to 5.8	0.64	-1.8	-9.4 to 5.8	0.64
> 1 hour	0.1	-9.7 to 9.9	0.98	0.3	-9.5 to 10.1	0.96	0.3	-9.5 to 10.1	0.95
Computer									
0 hours	ref	ref	ref	ref	ref	ref	ref	ref	ref
> 0 and ≤ 1 hour	-2.3	-11.7 to 7.1	0.63	-3.1	-12.4 to 6.3	0.51	-3.1	-12.5 to 6.3	0.52
> 1 hour	-11.3	-22.0 to -0.6	0.04	-11.7	-22.4 to -1.0	0.03	-11.7	-22.4 to -1.0	0.03

BMI, Body Mass Index; CI, confidence interval; NAPLAN, National Assessment Programme–Literacy and Numeracy; SDQ, Strengths and Difficulties.

^a^ Adjusted for corresponding media use (grade 3), child sex, child age (in years) (grade 3), Socio-Economic Indexes for Areas (SEIFA) advantage/disadvantage quintile (grade 3) and corresponding NAPLAN score (reading or numeracy) (grade 3).

^b^ Adjusted for corresponding media use (grade 3), child sex, child age (in years) (grade 3), Socio-Economic Indexes for Areas (SEIFA) advantage/disadvantage quintile (grade 3), SDQ total score (grade 3) and corresponding NAPLAN score (reading or numeracy) (grade 3).

^c^ Adjusted for corresponding media use (grade 3), child sex, child age (in years) (grade 3), Socio-Economic Indexes for Areas (SEIFA) advantage/disadvantage quintile (grade 3), SDQ total score (grade 3), BMI z-score (grade 3) and corresponding NAPLAN score (reading or numeracy) (grade 3).

## Discussion

To our knowledge, this is the first large population-based study to examine the association between media use and academic performance across two points in time in childhood using a national test of achievement. For children who watched more than two hours of television per day at 8–9 years of age there was a modest decrease on reading performance at 10–11 years of age, corresponding to a delay of around four months in learning. In cross-sectional analyses, watching between one and two hours or two plus hours of television was associated with a decrease in reading performance. However, when controlling for prior media use, the associations between concurrent television use and reading were no longer apparent suggesting that it is cumulative rather than concurrent media use which is associated with declines in reading performance. Watching more than two hours of television per day in grade 5 was also associated with lower numeracy even after controlling for prior media use, equivalent to the loss of a third of a year’s learning. Using a computer for more than an hour was associated with a lower numeracy performance, an association that again remained after control for prior media use, and equivalent to a loss in learning of around four months. These patterns of association remained similar after controlling for mental health difficulties and BMI. We found no relationship between video game use and academic performance.

Our results extend previous studies. In a study of 14-24-year-olds, television viewing was negatively associated with academic performance but internet use and game playing showed no association [31]. However, self-report of academic achievement was used rather than objective measurement, as in our analyses using linked academic data. Our findings also suggest that the negative effects of long-term television viewing are consistent across reading but not numeracy. One reason for these findings may be that television viewing displaces activities such as homework, reading, physical activity and sleep [14, 52]. Television viewing in childhood (ages 5–11 years) has been linked with increased attentional problems in adolescence (13 and 15 years of age) [53] and insufficient sleep has also been linked with deficits in academic performance [16]. Higher levels of television viewing have also been shown to affect the prefrontal cortex (frontopolar), which is associated with intellectual ability in children and adolescents [22]. Also, in the current study, participants spent between two to three times longer watching television compared with playing video games or using computers, which may explain our findings of associations between television viewing and academic performance. Television viewing may be a marker of a less stimulating home environment [54], which is associated with poorer academic performance [55].

Higher levels of computer use were associated with poorer numeracy performance but not reading performance. Existing evidence examining the effect of computer use on academic performance is mixed. Some studies suggest it may have beneficial effects [25]; for example, using a computer may allow children to access the internet, work on homework and connect with friends and family. But other studies suggest there is no impact [30, 56]. The discrepancies between studies and our conflicting findings between numeracy and reading performance may in part result from how computers were being used. Unfortunately, media content was not captured in the current study. Some evidence from cognitive neuroscience suggests stronger links between early attention and mathematical skills than early attention and reading skills [57]. Computer use may disrupt attention, which in turn impacts performance in numeracy [58, 59].

We found no association between video games and academic performance, which is in line with some previous research in adolescents [30]. However, some studies suggest that playing video games may be beneficial, for example by helping children learn how to problem solve [60], although this may depend on the type of games being played. The reasons why we did not find an effect of video game playing might include the relatively low levels of use in this sample and it is possible that parents regulate use in this young sample. It has also been suggested that young people who play video games regularly may habituate to the activity, thereby reducing the impact on academic outcomes [30].

The strengths of this study include the large sample size and longitudinal design, narrow age range focused in late childhood, measures of several types of electronic media and linkage with a standardised assessment of achievement. Several limitations also warrant noting. An active parental consent process was employed at recruitment with only 54% of parents providing written consent and the sample was skewed towards higher SES. Parents reported on their child’s media use with the possibility of misreporting. However, there is evidence that in this age group parents are reliable informants [25]. When controlling for BMI and emotional and behavioural problems in the current study, the patterns of association remained similar. However, it is possible that other unmeasured variables, such as parenting style (which is associated with media use [61] and academic performance [62] in late childhood and early adolescence), may also influence these findings. The current study utilised a device-based categorisation of time spent using media. However, the nature of screen time is changing with the rise of portable and accessible devices. Furthermore, media use is increasingly fragmented and multitasked making it increasingly difficult to estimate time spent using various media. This study did not include a measure of smartphone use but given participants were 11 years of age and under, rates of smartphone use are fairly low with around one in four 8–11 year-olds owning their own phone according to a recent national survey [63].

## Conclusion

In one of the first longitudinal studies to examine the relationship with academic performance in late childhood, we found that heavy television use predicted a loss of reading of four months relative to peers two years later: heavy computer use predicted a similar loss in numeracy two years later. The results suggest that it is likely to be cumulative heavy television and computer use that should be a focus in framing responses from teachers and parents, as well as broader education systems. Given that media use commonly increases in the transition to adolescence, researchers should now turn to estimate the cumulative effects of longer term media exposures on educational attainment in later secondary school.

## Supporting information

S1 Table Details of the multiple imputation model used to generate imputed data for the investigation of the short-term and long-term effects of media use on academic performance. (DOCX)
