# Supplementary material for: Electronic media use and academic performance in late childhood: A longitudinal study
Source: PLoS One. 2020 Sep 2;15(9):e0237908. doi: 10.1371/journal.pone.0237908 (PMC7467319; doi:10.1371/journal.pone.0237908)
Supplement: S1 Table — (DOCX) [file pone.0237908.s001.docx]

| Supplementary Table 1: Details of the multiple imputation model used to generate imputed data for the investigation of the short-term and long-term effects of media use on academic performance. | | | |
| --- | --- | --- | --- |
| **Variable description** | **Missing data (%)** | **Regular or imputed variable** | **Analysis or auxiliary variable** |
| Age (centred about the mean) (wave 1) (continuous) | 0.0 | Regular | Analysis (covariate) |
| SEIFA ad/disad quintile (wave 1) (ordinal) | 0.0 | Regular | Analysis (covariate) |
| Gender (binary) | 0.0 | Regular | Analysis (covariate) |
| School id (wave 3) (categorical) | 0.0 | Regular | Auxiliary |
| School sector (wave 1) (categorical) | 0.0 | Regular | Auxiliary |
| BMI z-score (wave 1) (continuous) | 4.3 | Imputed | Analysis (covariate) |
| BMI z-score (wave 2) (continuous) | 7.3 | Imputed | Auxiliary |
| SDQ total score (wave 1) (continuous) | 2.8 | Imputed | Analysis (covariate) |
| SDQ total score (wave 2) (continuous) | 29.3 | Imputed | Auxiliary |
| NAPLAN reading score (wave 1) (continuous) | 13.9 | Imputed | Analysis (covariate) |
| NAPLAN reading scaled score (wave 3) (continuous) | 16.5 | Imputed | Analysis (outcome) |
| NAPLAN numeracy scaled score (wave 1) (continuous) | 15.0 | Imputed | Analysis (covariate) |
| NAPLAN numeracy scaled score (wave 3) (continuous) | 16.5 | Imputed | Analysis (outcome) |
| Watching TV ( average hours/day) (wave 1) (ordinal) | 25.8 | Imputed | Analysis (exposure or covariate) |
| Watching TV( average hours/day) (wave 2) (ordinal) | 29.2 | Imputed | Auxiliary |
| Watching TV (average hours/day) (wave 3) (ordinal) | 28.8 | Imputed | Analysis (exposure) |
| Playing video games (average hours/day) (wave 1) (ordinal) | 25.8 | Imputed | Analysis (exposure or covariate) |
| Playing video games (average hours/day) (wave 2) (ordinal) | 29.3 | Imputed | Auxiliary |
| Playing video games (average hours/day) (wave 3) (ordinal) | 31.5 | Imputed | Analysis (exposure) |
| Using a computer (average hours/day) (wave 1) (ordinal) | 25.8 | Imputed | Analysis (exposure or covariate) |
| Using a computer (average hours/day) (wave 2) (ordinal) | 29.2 | Imputed | Auxiliary |
| Using a computer (average hours/day) (wave 3) (ordinal) | 31.6 | Imputed | Analysis (exposure) |
| Teacher rating of student’s ability in English (wave 1) (ordinal) | 7.8 | Imputed | Auxiliary |
| Teacher rating of student’s ability in mathematics (wave 1) (ordinal) | 7.8 | Imputed | Auxiliary |
